# Load distribution between cephalic screws in a dual lag screw trochanteric nail

**DOI:** 10.1186/s13018-016-0377-y

**Published:** 2016-04-01

**Authors:** Julia Henschel, Sebastian Eberle, Peter Augat

**Affiliations:** Institute of Biomechanics, Trauma Center Murnau, Murnau, Germany; Paracelsus Medical University, Salzburg, Austria

**Keywords:** FEM, Cut-out, Z-effect, Dual cephalic screw implant

## Abstract

**Background:**

It has been observed clinically that the Z-effect is a potential cause of failure of an intramedullary nail with two cephalic screws. It describes the migration behavior of the cephalic screws in the femoral head. The primary objective was to examine different cephalic screw configurations and test the load distribution between them as a function of their relative placement and their relative movement in the nail. It has been hypothesized that different cephalic screw positions may have an influence on the stress in the implant and bone and therefore on implant failures, such as the Z-effect.

**Methods:**

To quantify the load distribution of a dual cephalic screw intramedullary femoral nail (Citieffe, Calderara di Reno, BO, Italy), a finite element model of the femur, focusing on the loading of the cephalic screws, was prepared. Four different screw lengths (90–105 mm) were examined. The investigation considered the stresses and strains in the bone and implant as well as the relative movement of the screws.

**Results:**

If the inferior cephalic screw had a shorter length, then the superior one and the femoral nail had to bear higher loads. In that case, the “equivalent von Mises stress” increased up to 10 % at the superior cephalic screw and up to 5 % at the femoral nail. The analysis of the relative movement showed that sliding of the inferior cephalic screw occurred in the nail. The total movement ranged from 0.47 to 0.73 mm for the different screw configurations.

**Conclusions:**

The stresses were distributed more equally between the two cephalic screws in the bone and the implant if a longer inferior screw was used. The stresses in the bone and implant were reduced with a longer inferior cephalic screw. Therefore, a configuration using a longer inferior cephalic screw is preferable for trochanteric fracture fixation with a dual cephalic screw intramedullary device.

## Background

The reported number of proximal femoral fractures has increased steadily and due to aging populations, a further increase can be expected [1, 2]. The dynamic hip screw (DHS) with a single cephalic screw is one of the most widely used implants for these fractures [3, 4], but complications like cut-out and rotation of the proximal fragment have been reported in unstable fracture situations or osteoporotic bone [5, 6]. Recent studies recommended that it is preferable to use an intramedullary instead of an extramedullary fixation in trochanteric fractures [7, 8]. The use of an intramedullary nail is associated with shorter operative time, less blood loss, and higher failure load of an implant. It has also been documented in literature that an intramedullary nail is to be preferred in unstable fractures [9, 10].

Intramedullary devices for the fixation of proximal femur fractures are available with single, dual, and two integrated lag screws. Clinical experience with single and integrated screw design is most favorable for intertrochanteric fractures and subtrochanteric fractures. Dual lag screw designs may have a role for medially located trochanteric fractures or lateral fractures of the femoral neck and have demonstrated sufficient mechanical stability [6, 11]. However, dual cephalic screw implants may occasionally fail through the so called Z-effect [11–13] in which there is an opposite migration of the two cephalic screws during post-operative weight-bearing [12], with medial migration of the superior and lateral migration of the inferior cephalic screw. An opposite migration pattern, in which the superior screw moves laterally and the inferior screw medially, has been described as the reversed Z-effect [14].

One important factor affecting failure of implants in the femoral head is their localization within the head. For extramedullary implants, the tip to apex distance (TAD) has been identified as an important factor predicting cut-out through the femoral head. It has been shown that cut-out is more likely with an increasing value of TAD [15–17].

Also for intramedullary implants, the location of the cephalic screw within the femoral head has been suggested as an important factor influencing the failure of implant fixation [18, 19]. These findings cannot directly be transferred to dual screw designs. However, it appears likely that the screw position in the femoral head and the relative placement of the two screws affects the fixation of the osteosynthesis devices. The load transfer from the bone to the screws and then to the intramedullary nail might be changed by altering the relative positions of the cephalic screws. This could potentially affect implant failure, cephalic screw cut-out, or the occurrence of screw migration, including the Z-effect.

Therefore, the hypothesis of our study was that the cephalic screw positions in a dual cephalic screw design affect the load distribution in the implant and the surrounding bone. The specific aims of this computational analysis were to determine the local stress distributions and micro-movements under load application for different cephalic screw configurations.

## Methods

To quantify the load distribution, a finite element (FE) analysis of the proximal femur was prepared with emphasis on the loading of the two cephalic screws in a cephalomedullary implant for proximal femur fracture fixation. The model comprised a proximal fracture of the femur fixed with an EBA^2^ intramedullary femoral nail (Citieffe, Calderara di Reno, BO, Italy). The reaction loads at the support of the cephalic screws in the nail, and the maximum strain/stress on the cephalic screws as a response to the applied hip joint force and the fixation of the shaft, were both assessed for different positions of the cephalic screw tips.

The study was divided into two parts. In the first part, a mechanically stable configuration representing ideal repositioning of the fracture fragments was modeled. In the second part, an unstable configuration representing inadequate repositioning, reduced bone quality, and sliding of the cephalic screw was investigated.

### Stable configuration

The FE model in this study was based on a standardized femur geometry with homogenous bone material properties for cortical and cancellous bone (compressive strength of cortical bone 157 MPa, compressive strength of cancellous bone 6.0 MPa, Poisson’s ratio of cortical bone 0.26, Poisson’s ratio of cancellous bone 0.3) which has been previously described in detail [20]. The model included an unstable trochanteric fracture (AO Type 31 A2.2) [21, 22] fixed by the EBA^2^ nail system. The implant was inserted according to the surgical manual, with no interfragmentary gap. The implant material was modeled as Titanium Alloy Ti6Al4V with material constants: Elastic Modulus E [MPa] 115,000; Poisson’s ratio *μ* 0.342.

The boundary conditions of the FE model mimicked a physiological load in stance. The knee joint was simulated as a cardanic joint and the hip as a ball and socket joint. A force of 1900 N was applied at the center of femoral head, which represents the physiological load of the hip joint during walking for an 80 kg person [23].

The model of the femur including the fracture and the intramedullary nail was created using FE software (Design Modeler®, ANSYS® Academic Research, Release15.0, ANSYS, Inc., Canonsburg, PA, USA). Full models were designed with different contact types to determine which contact behavior was the most suitable. For further studies, the “simplified friction multipoint constraint contact (MPC)” model was used. This means that the threads of the cephalic screws are simplified and bonded by a multipoint constraint formulation. Thus in the stable configuration simulation, no sliding of the cephalic screws could occur in the proximal fragment of the bone. All FE analyses were conducted with ANSYS (ANSYS® Academic Research, Release 15.0, ANSYS, Inc., Canonsburg, PA, USA).

#### Validation

For validation purposes, the FE model of the stable trochanteric fracture was constructed as a mechanical model using a composite adolescent-sized femur (model 3406, Sawbones, Malmoe, Sweden). The simulated models were validated by four mechanical tests. One test was performed as a reference on the intact surrogate femur. Three further tests used different lengths of cephalic screws (superior 105 mm, inferior 100 mm; superior 105 mm, inferior 90 mm; superior 90 mm, inferior 105 mm).

An experienced surgeon performed the surgery and created a fracture with a bone saw. The femoral head was embedded in a hemisphere with casting resin (RENCAST FC53, Huntsman Advanced Materials GmbH, Bergkamen, Germany) to ensure adaption to the test machine. Thereby, the hemispherical shape represented the hip-like ball joint. The distal part of the femur was embedded to fit into a custom made cardanic joint. The mechanical tests were conducted with a servo-electric testing machine (Zwick 010, Zwick, Ulm, Germany). A quasi-static axial compression test with a load ramp up to 1000 N was performed. For the validation, the experimental and simulated results were compared for axial stiffness, bone deformation, and strains at the bone and the implant.

The strains were measured by strain gauges (SG) (KFG-1-120-C1-11L3M3R, Kyowa, Tokyo, Japan). The bone was fitted with four SGs. One SG was fixed superiorly and one inferiorly on the femoral neck. The other two SGs were fixed medially and laterally on the femur shaft. The EBA^2^ nail was fitted with four SGs: one posteriorly above the cephalic screw holes and one anteriorly below the screw holes. Additionally, one SG was fixed at every cephalic screw. The SGs were coupled to an amplifier (Spider 8, HBM, Darmstadt, Germany). The recording and the evaluation of the data was performed with additional software (Catman easy, HBM, Darmstadt, Germany). Local displacements were detected by optical markers on the bone surface and an optical tracking system (Pontos, GOM, Braunschweig, Germany) which recorded the displacements of optical markers on the bone surface with an accuracy of 0.025 mm.

#### Parameter study

The primary objective of the study was to examine different cephalic screw configurations and determine the load distribution between the two cephalic screws in the EBA^2^ femoral nail as a function of their relative placement. Strains and stresses were examined at the bone and the implant with emphasis on the stresses at the cephalic screws and in the surrounding bone. The stresses were recorded from the straight part of the screw before the chamfer, and the relative movements of the cephalic screws within in the nail were investigated. Four different insertion lengths with a range of 90 to 105 mm were examined for each cephalic screw, so there was a total of 16 different screw configurations.

### Unstable configuration

The computational model for the unstable configuration was an extended version of the validated model of the stable configuration. Compared to the stable model, it contained modification of the fracture, the bone quality, and the screw geometry.

**Fig. 1:**
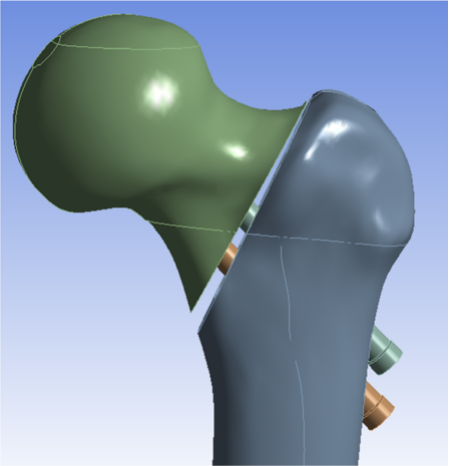
Fracture gap without bone contact

The unstable model was created with a fracture without any bone contact (Fig. 1). The fracture gap remained open in the loaded and unloaded cases. For that reason, the initial support between the head and shaft fragments was only through load transfer through the screws and not through bone contacts.

The quality of the bone was changed. In the unstable model, the Young’s modulus of the cancellous bone was reduced to 80 MPa, which represented a bone with low bone quality. The unstable fracture with low bone quality represented a worst-case scenario.

While in the stable model a simplified screw model without threads was used, the unstable model included simplified circular threads. Additionally, the contact between the screw and the bone was changed. This allowed for the screws to slide with a resistance proportional to the frictional coefficient of *f* 0.08 [24]. This contact type simulated realistic behavior relating to an unstable fracture.

## Results

The examined stresses are equivalent von Mises stresses. Only partially and specifically indicated normal stress was examined at the screws.

### Stable configuration

The percentage error between numerical and experimental result was largest for the strain measurements (−36 % ± 27 % (mean ± SD)) and smallest for measurements of axial stiffness (−10 % ± 12 %). While strain and stiffness appeared to be underestimated by the FE analysis, local displacements were overestimated by about 25 % ± 21 %.

#### Stresses at the implant

**Fig. 2:**
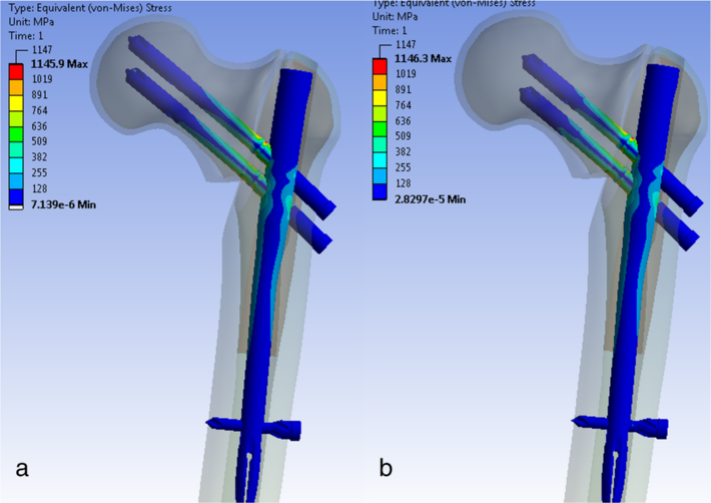
“Equivalent von Mises stresses” for two different screw configurations. Superior screw 105 mm, inferior screw 105 mm (**a**). Superior screw 90 mm, inferior screw 90 mm (**b**). Highest stress levels observed at the superior aspect of the screw (1136 MPa) and at the superior site of the screw hole in the nail (1146 MPa) for both configurations

Comparing the stress on the implant including the cephalic screws, it could be observed that across all combinations of screw lengths, the difference between the stresses in the implant was less than 3 % (Fig. 2).

**Fig. 3:**
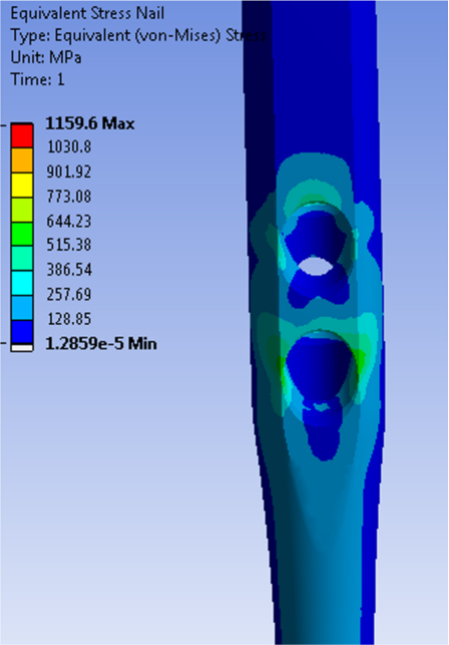
“Equivalent von Mises stresses” around the bore hole of the femoral nail

The maximum stress on nail was always observed in the area around cephalic screw holes of the intramedullary nail (Fig. 2). The stresses were largest on the lateral side of the hole and lowest at the inferior side (Fig. 3).

The largest stresses on the screws occurred around the chamfer and reached a maximum of 1168 MPa at the superior screw and 1078 MPa at the inferior screw (Fig. 2). On average, the superior screw carried 9 % more load than the inferior screw. This did not depend on the configuration of the cephalic screws.

#### Stresses in the bone

**Fig. 4:**
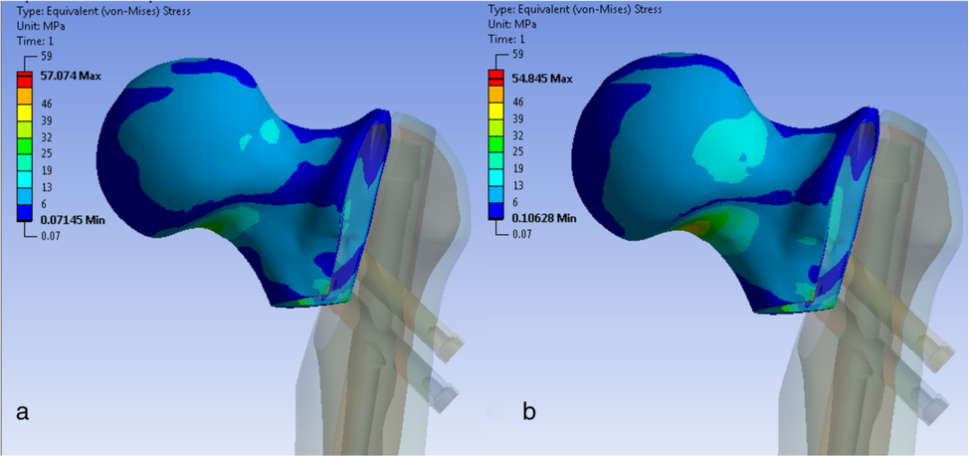
“Equivalent von Mises stresses” in the cortical bone of the proximal fragment for two different screw configurations. Superior screw 105 mm, inferior screw 105 mm (**a**). Superior screw 90 mm, inferior screw 90 mm (**b**). Indicated are the stress values at the inferior cortex of the femoral neck (**a** 35 MPa, **b** 50 MPa)

The stress in the cortical bone of the femoral neck changed for different cephalic screw configurations. With decreasing length of the inferior cephalic screw, the stress increased, especially in the inferior cortex and also at the anterior aspect of the transition between neck and head (Fig. 4).

**Fig. 5:**
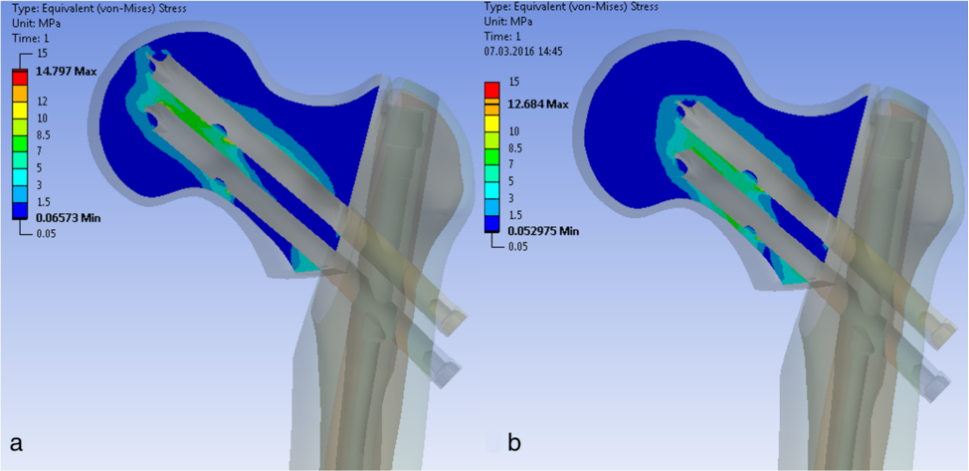
“Equivalent von Mises stresses”—cancellous bone. Superior screw 105 mm, inferior screw 105 mm (**a**). Superior screw 90 mm, inferior screw 90 mm (**b**). Indicated are the stress values between the cephalic screws (**a** 6.7 MPa, **b** 6.5 MPa)

The stress maxima in the cancellous bone of the proximal fragment were found in between and above the two cephalic screws. As the length of the screws decreased, the maxima shifted from the head to the neck area (Fig. 5). Another stress maximum occurred at the contact area of the proximal head fragment and the femoral shaft. In addition, the cancellous bone beneath the inferior cephalic screw at the inferior location of the head-neck fragment experienced compression. The average stress between the cephalic screws was 6.6 MPa. There were differences in the stress in the cancellous bone for the cephalic screw configurations. These differences were relatively small, remaining below 3 %.

### Unstable configuration

#### Stresses at the implant

**Fig. 6:**
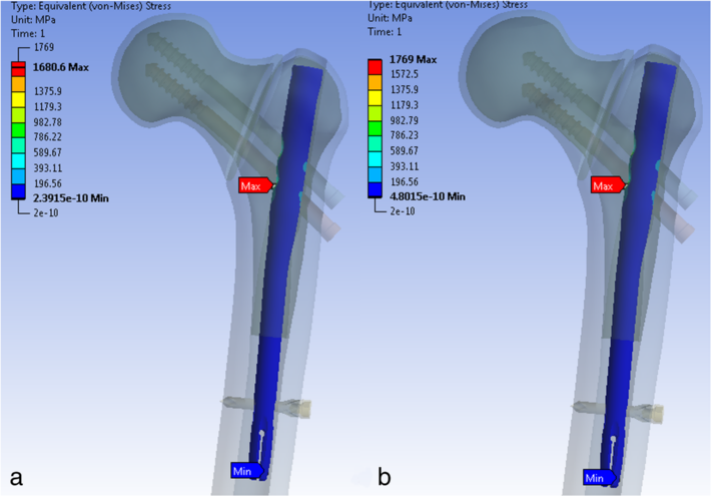
“Equivalent von Mises stresses” on the femoral nail for the unstable fracture situation. Superior screw 105 mm, inferior screw 105 mm (**a**). Superior screw 90 mm, inferior screw 90 mm (**b**)

The stress distribution at the nail in the unstable configuration was similar to that of the stable fracture situation. The stress maxima were located around the bore holes and reached values of up to 1748 MPa at the nail (Fig. 6). In the nail, the stress increased as the length of the inferior screw became shorter. Thus with decreasing screw length, the stress was transferred from the inferior screw to the nail where it manifested at the screw hole. For the different configurations, the stresses in the nail differed by less than 5 %.

**Fig. 7:**
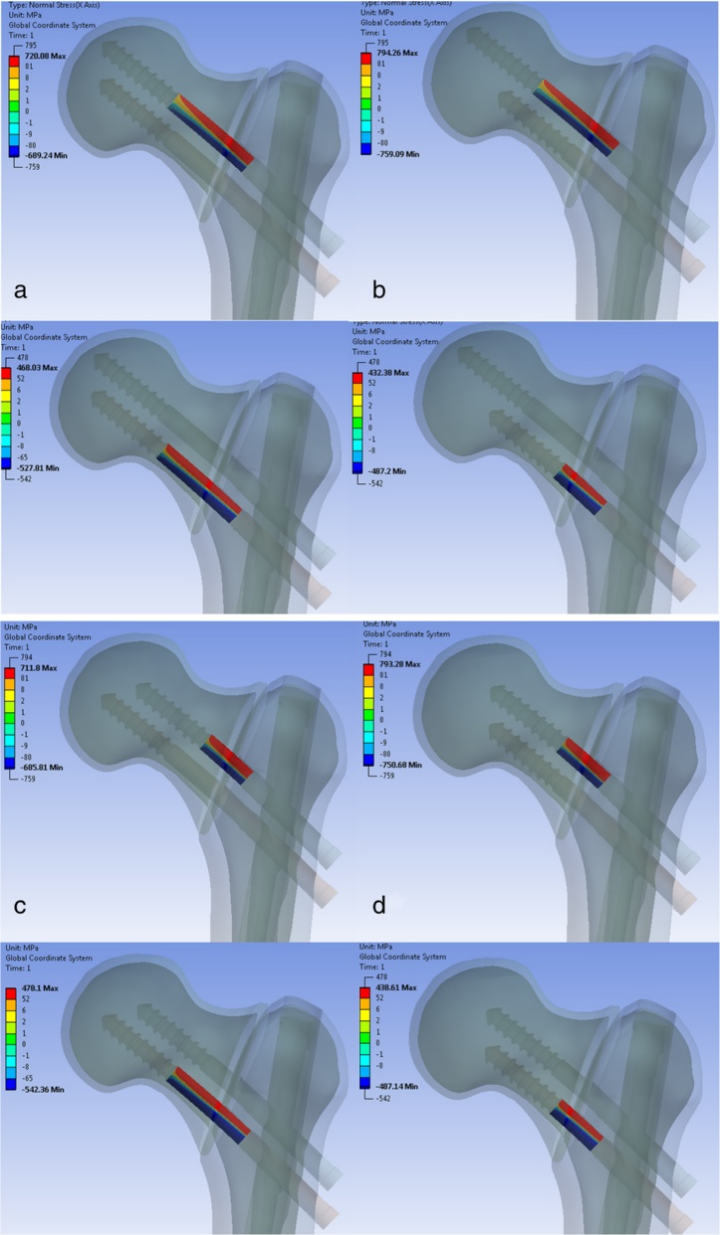
Normal stress at the two cephalic screws for four different extreme screw configurations. Superior screw 105 mm, inferior screw 105 mm (**a**). Superior screw 105 mm, inferior screw 90 mm (**b**). Superior screw 90 mm, inferior screw 105 mm (**c**). Superior screw 90 mm, inferior screw 90 mm (**d**)

The maximum stress in the cephalic screws occurred at the posterior surfaces of both screws. With a shorter inferior screw, the superior cephalic screw had to bear higher loads. This resulted in increased tensile normal stress for the superior screw and reduced compressional normal stress for the inferior screw (Fig. 7).

Therefore, the normal stresses were the highest at the superior screw for the 105/90 configuration at 794 MPa and for the configuration with the smallest screws (90/90) at 793 MPa. For those two configurations, the stresses at the inferior cephalic screws were lowest. The normal stresses at the inferior screw were 432 MPa for the 105/90 configuration and 439 MPa for the 90/90 configuration.

The stress difference between the superior and the inferior screw was smallest for configurations with longer inferior screws. The normal stress difference was 252 MPa for the configuration with the longest screws and 233 MPa for the 90/105 configuration.

#### Stresses in the bone

**Fig. 8:**
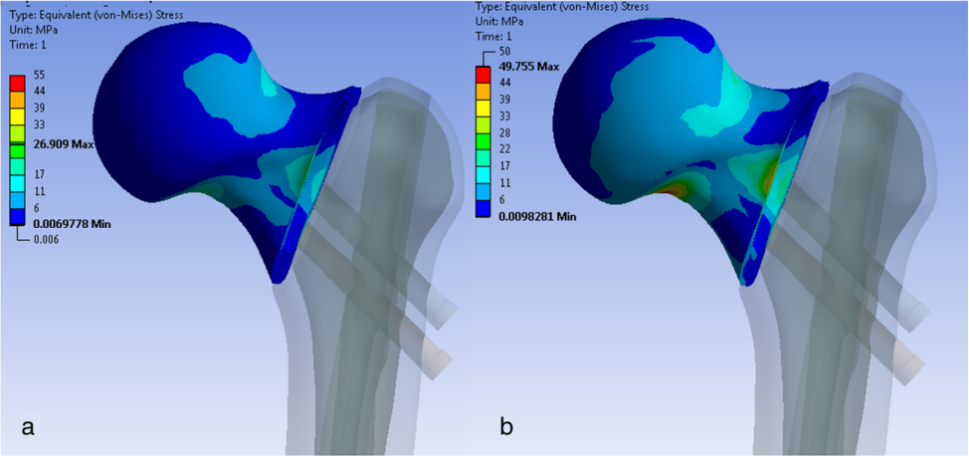
“Equivalent von Mises stresses” in the cortical bone of the proximal fragment for two different screw configurations. Superior screw 105 mm, inferior screw 105 mm (**a**). Superior screw 90 mm, inferior screw 90 mm (**b**). Indicated are the stress values at the inferior cortex of the femoral neck (**a** 27 MPa, **b** 50 MPa)

Similar to the stable configuration, stress in cortical bone concentrates in the inferior location of the neck to head region. The stress increases when the length of the inferior cephalic screw decreases (Fig. 8).

**Fig. 9:**
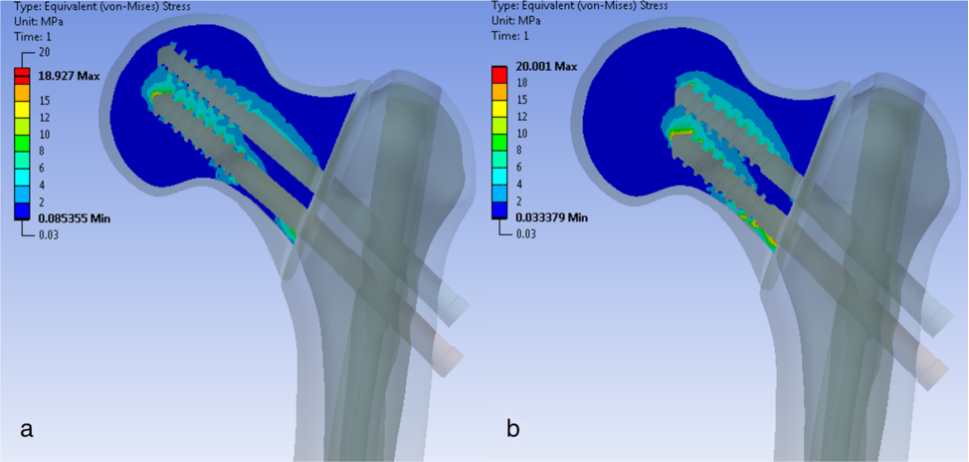
“Equivalent von Mises stresses”—cancellous bone. Superior screw 105 mm, inferior screw 105 mm (**a**). Superior screw 90 mm, inferior screw 90 mm (**b**). Indicated are the stress values between the cephalic screws (**a** 4.6 MPa, **b** 6.5 MPa)

The stress distribution within the cancellous bone is similar for the stable and unstable configurations. The stress maxima occurred between the two screws and above them (Fig. 9). However, with decreasing length of the screws, the absolute value of the stress considerably increased by up to 40 % (Fig. 9).

### Relative movement of the screws within the nail

**Fig. 10:**
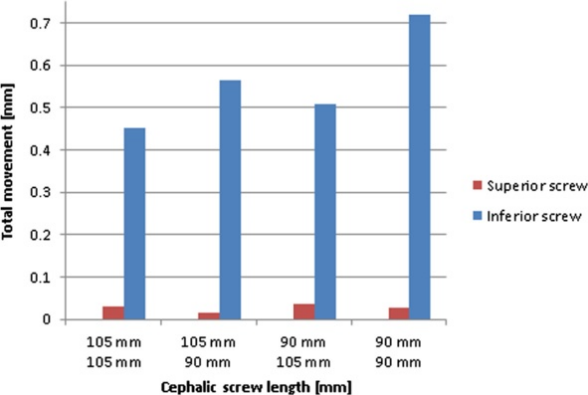
Sliding of the screws in the nail in lateral direction

The relative movement of the screws within the nail was calculated to determine possible sliding of the screws. A lateral sliding of the inferior screw was observed for all screw configurations. The largest sliding of the inferior screw was 0.7 mm. This was observed in the configuration with both screws having the smallest length (90 mm/90 mm). In contrast, the sliding of the superior screw remained below 0.03 mm for all of the configurations (Fig. 10).

## Discussion

The objective of this study was to analyze the effects of using different configurations of lag screw placement on the mechanical behavior of a dual lag screw trochanteric nail. Our findings indicate that by using a longer inferior lag screw, stress within the bone, the nail and the superior lag screw can be reduced and the difference in stress between the superior and inferior screws was smallest with a longer inferior screw. The length of the superior screw had minor influence on the stress in the bone and within the implant. The analysis of screw displacement within the nail demonstrated a lateral migration of the inferior screw during loading.

Due to the fact that our study represents a FE study, the preferred cephalic screw configuration is a suggestion and has to be proved by further mechanical tests and clinical investigations. Computational models require simplifications of real-world situations and thus always have inherent limitations. Our FE model simulated only one type of fracture with two variations of bone density and two qualities of fracture reduction and therefore represents only a limited part of the large clinical variety of trochanteric fracture situations. The bone model used in the simulation was based on a simplified biomechanical bone model which has been previously validated [20]. While this model does not represent local variation in bone density and cancellous architecture, previous studies demonstrated that the model reproduces the flexural and torsional rigidity, and screw pull-out strength of human bone [25, 26]. The loading applied to the femoral head represented a characteristic load scenario during weight-bearing in full stance. It did not include further torsional or bending loads which could potentially occur during other activities of daily living like rising from a chair or squatting. Finally, the FE model only induced static loads and evaluated the elastic material response. Migration and cut-out of screws, however, is very likely to occur due to cyclic fatigue of bone [11]. Thus the analysis of peak elastic stress during static loading might only indicate the location of potential failure of bone material. In order to justify our FE model with respect to the loads in the implant and the strains on the bone surface, mechanical tests were performed on synthetic bone specimens demonstrating the validity of the FE model.

Two different FE models were used in this study to analyze implant loading for different lag screw configurations. While the stable model simulated an ideal fracture reposition in a healthy bone, the unstable model simulated a worst-case scenario with imperfect fracture reposition and reduced bone quality. In the stable model, the effect of different lag screw configurations on the load distribution in the implant bone construct was rather small. This would indicate that the treatment of trochanteric fractures is not sensitive to the placement of the lag screws within the limits investigated in this study. In the unstable model, the effect of cephalic screw placement on the load distribution of the implant bone construct became more pronounced. All of the parameters assessed in the FE study were affected by lag screw placement. The most dominant effects were found for screw displacement and stress within the bone.

The values of stress within the bone and the implant material can indicate potential locations of failure or fatigue. Stresses in the proximal bone fragment were largest at the inferior location of the femoral neck and reached values of up to 50 MPa. Although these stress values were still below the strength of femoral cortical bone (180–209 MPa) [27], the location of the stress concentration might explain the clinically observed occurrence of varus collapse [28]. The maximum stresses at the implant occurred around the cephalic screw holes of the nail. For the stable configuration, the maximum stress exceeded 1100 MPa and reached the yield strength of most titanium alloys [29]. Based on the results of our mechanical validation data, the local deformations and thus the strains and stresses were slightly overestimated by the FE model. Therefore, the effective stresses at the nail are most likely somewhat lower, not leading to direct failure. Nevertheless, the location of these stresses at the inferior aspect of the cephalic screw holes in the nail is a frequent location of implant failure in cephalomedullary devices [28, 30].

In our study, the lateral sliding of the inferior lag screw during loading is consistent with the description of the clinically observed Z-effect in dual lag screw implants, in which the inferior lag screw migrates laterally, and the superior lag screw migrates medially during physiologic loading. The lateral sliding was largest if the inferior screw had the shortest length. This indicates that a short insertion depth of the inferior screw would support the occurrence of the Z-effect. It has to be noted, however, that in this study the model was tested under a static load, while the Z-effect is assumed to be a phenomenon which occurs under cyclic loading [11]. The clinical recommendation to prevent the Z-effect by inserting of the inferior cephalic screw as close as possible to the inferior cortex of the femoral neck [31] would be supported by our findings.

## Conclusions

In conclusion, our results demonstrate that the configuration of cephalic screw placement in dual cephalic screw implants influences the load distribution at the implant and the bone. The size of the effect depends on the stability of the osteosynthesis and becomes more relevant in osteoporotic bone and imperfect fracture reduction. The most favorable load distribution with reduced overall stress was achieved using longer inferior cephalic screws. This could potentially decrease the likelihood of implant failure, varus collapse, and occurrence of screw migration. These biomechanical findings confirm clinical recommendations for this implant type.
